# Invasive angiolipoma of the mediastinum and lung

**DOI:** 10.1136/thorax-2024-222337

**Published:** 2025-02-12

**Authors:** Ziyin Shang, Yongjie Luo, Xiaoling Kang, Chun Hong, Yuan Si

**Affiliations:** 1 Department of Pediatric Thoracic Surgery, Guangdong Women and Children Hospital, Guangzhou, China; 2 Department of Pathology, Guangdong Women and Children Hospital, Guangzhou, China; 3 Department of Neurology, Third Affiliated Hospital of Sun Yat-Sen University, Guangzhou, Guangdong, China

**Keywords:** Child, Paediatric Lung Disaese, Rare lung diseases, Thoracic Surgery, Bronchoscopy

**Figure 1:**
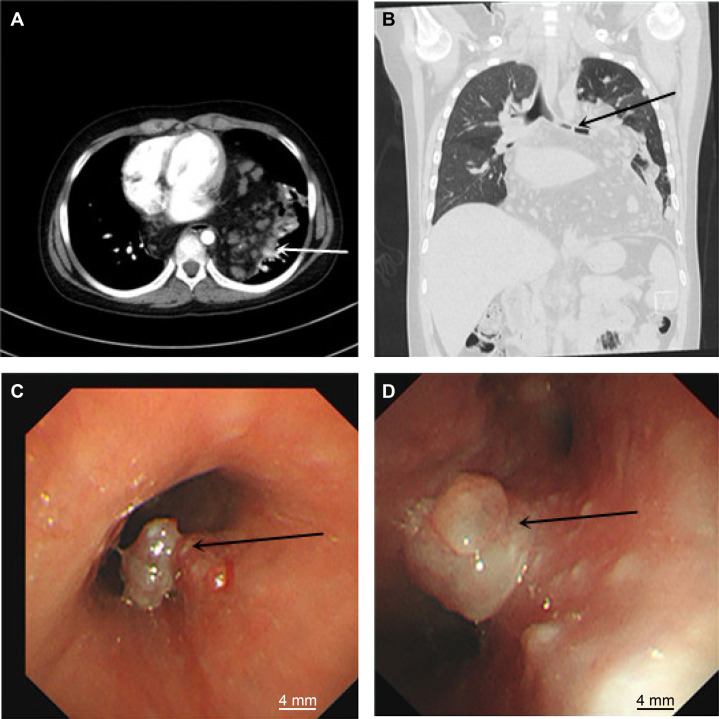
(**A**) Axial CT scan of the chest showing the tumour encasing the mediastinal structures and protruding into both lung fields (white arrow). (**B**) Coronal CT scan of the chest showing the tumour involving the left lung and bronchus along the vascular spaces (black arrow). (**C**) Bronchoscopy showing a tumour obstructing the airway at the left main bronchus and carina between the left upper and lower lobes (black arrow). (**D**) Bronchoscopy showing a tumour obstructing the airway at the left upper and lower lobes (black arrow).

**Figure 2:**
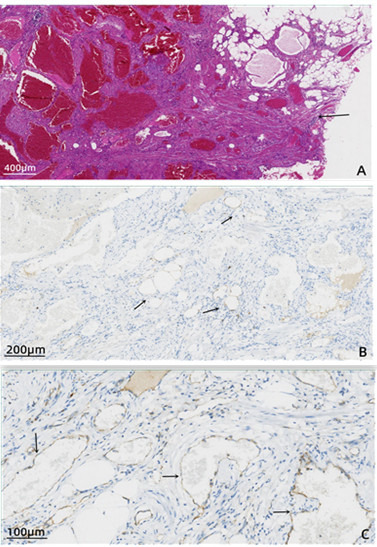
(**A**) Postoperative histopathological examination showing the tumour composed of vascular, adipose and fibrous tissues (HE, ×40 total magnification). The tumour is borderless and invades the surrounding smooth muscle tissue (black arrow). (**B**) Adipocyte positivity is shown using S100 (IHC, 100×total magnification). IHC, immunochemistry.

 A 12-year-old boy presented with a 1 month history of cough and exertional dyspnoea. Physical examination revealed a heart rate of 106 beats/min, a respiratory rate of 28 breaths/min and an O_2_ saturation of 90–95%. Bilateral breath sounds were coarse with audible rhonchi. Contrast-enhanced chest CT revealed a large mass in the mediastinum and left lung. The mass encased mediastinal structures and protruded into both lung fields, involving the left lung and bronchus along the vascular spaces (figure 1A, B). Bronchoscopy showed a tumour obstructing the left main bronchus and carina, with airway constriction due to external pressure (figure 1C, D). The tumour’s broad base, irregular margins and bleeding tendency prevented tissue biopsy. Angiolipoma is often diagnosed via imaging findings but must be differentiated from adipose-derived tumours, such as liposarcoma and adipoblastoma. For tumour debulking to control tumour compression symptoms and clarifying the pathology to guide further treatment, the patient underwent a left thoracotomy. During the operation, fatty changes were observed on the surface of the tumour, with multiple dark purple irregular protrusions and vasodilation. The tumour was attached to the left lung and diaphragmatic surface, and deep exploration indicated that the tumour had crossed the midline. Most of the mediastinal tumour was removed, and part of the lung tumour was biopsied. Postoperative histopathological examination revealed that the tumour was composed of vascular, adipose and fibrous tissues (figure 2A, B). Immunohistochemistry analysis showed positive results for CD31 (vascular +), CD34 (vascular +), desmin (+), S100 (adipocytes +), SMA (+) and Ki-67 (3%). The patient was diagnosed with angiolipoma of the mediastinum and lung. The patient’s shortness of breath improved postoperatively, and he was discharged 7 days postoperatively. We recommended endobronchial resection of the bronchial tumour at 1 month postoperatively, although the parents refused. The patient did not receive radiotherapy and was not followed up.

Angiolipoma is a rare benign soft tissue tumour, which is divided into invasive and non-invasive angiolipoma, with invasive angiolipoma characterised by the lack of an envelope and infiltration into surrounding tissues. In our patient, the tumour originated from the mediastinum, infiltrating along the vascular spaces into the left lung, pleura and bronchus. This case underscores the invasive nature of the disease. The main treatment options for invasive angiolipoma are surgical resection and transarterial embolisation.^1^ There is a current controversy over whether radiotherapy should be performed after surgery. Some scholars believe that radiotherapy after surgical resection can reduce the recurrence rate,^2^ while others argue that it will cause unnecessary side effects. However, postoperative recurrence is rare even if complete resection is not possible.^3^ In this patient, no obvious cell atypia and nuclear mitosis were found by pathology. Ki-67 was approximately 3%, indicating low proliferative activity of tumour cells. Considering the potential side effects of radiotherapy and the risk of causing more harm than benefit in children, radiotherapy was not used.
